# The Contralateral Lung: An Opportunity for Lung Transplant at a Lower Lung Allocation Score?

**DOI:** 10.1016/j.atssr.2024.11.001

**Published:** 2024-11-13

**Authors:** Emily L. Larson, Jessica M. Ruck, Alexandra Rizaldi, Alice L. Zhou, Alfred J. Casillan, Jinny S. Ha, Christian A. Merlo, Errol L. Bush

**Affiliations:** 1 Division of Thoracic Surgery, Department of Surgery, Johns Hopkins School of Medicine, Baltimore, Maryland; 2 Division of Pulmonology, Department of Medicine, Johns Hopkins School of Medicine, Baltimore, Maryland

## Abstract

**Background:**

In more than half of single-lung transplants (SLTs), the second or contralateral lung is discarded. We evaluated the use of the contralateral lung and its role in enabling single-lung transplantation at a lower Lung Allocation Score (LAS).

**Methods:**

We identified adult SLT recipients from 2015 to 2022 using the United Network for Organ Sharing/Organ Procurement and Transplantation Network database. Post-SLT survival was compared with Kaplan-Meier curves and multivariable Cox regression.

**Results:**

Of 4692 SLT recipients, 1246 (26.6%) received a contralateral lung. Contralateral lung recipients had a lower median LAS (36.9 [33.5-42.2] vs 40.4 [35.3-49.8]; *P* < .001) and higher median sequence number (26 [11-65.8] vs 8 [3-25]; *P* < .001). Survival was higher in contralateral vs first SLT recipients (median survival, 5.0 [95% CI, 4.7-5.3] years vs 4.5 [95% CI, 4.2-4.7] years; log-rank *P* < .001). This difference persisted as a 14% decreased hazard of mortality for contralateral recipients after adjustment (adjusted hazard ratio, 0.86 [0.77-0.95]; *P* = .004). Of SLT donors, 1309 (37.4%) had both lungs accepted for use.

**Conclusions:**

In this nationwide analysis, we found that contralateral SLT recipients received SLTs at a lower median LAS without compromising outcomes, suggesting that contralateral single-lung transplantation may provide a valuable approach to help expand the donor pool.

**In Short**

▪ In single-lung transplantation, low use of the contralateral lung provides an opportunity to expand the donor pool.
▪ Receipt of a contralateral vs first lung enabled single-lung transplantation at a lower Lung Allocation Score.
▪ Receipt of a contralateral vs first lung was associated with significantly improved survival.

Single-lung transplants (SLTs) account for nearly 20% of all lung transplantations performed in the United States^1^^,^^2^ However, in approximately 57% of donors whose organs are used for single-lung transplantation, only 1 lung from the donor is used, and the second or contralateral lung is discarded.^3^

The demand for SLTs far exceeds current availability, so identifying opportunities to expand the donor pool is crucial.^4^ The frequently unused contralateral lungs may represent such an opportunity for patients awaiting SLTs.^5^ Specifically, we hypothesized that recipients of a contralateral lung undergo single-lung transplantation at a lower Lung Allocation Score (LAS).

Outcomes of single-lung transplantation using contralateral vs first lungs are unknown. Thus, the objective of this study was to assess the outcomes of contralateral vs first SLT recipients and to determine whether acceptance of a contralateral lung enabled transplantation at a lower LAS.

## Material and Methods

This study was approved by the Johns Hopkins Medicine institutional review board (IRB00352819).

### Data Source

This study used data from the United Network for Organ Sharing/Organ Procurement and Transplantation Network (OPTN) database. This includes data of all donors, wait-listed candidates, and transplant recipients in the United States, submitted by the members of the OPTN.^6^ The Health Resources and Services Administration, US Department of Health and Human Services, provides oversight to the activities of the OPTN.

### Study Population

Adult (≥ 18 years) recipients of single lung–only transplants between January 2015 and December 2022 were retrospectively reviewed. Recipients were stratified by receipt of contralateral vs first lungs, determined by offer order.

### Donor, Recipient, and Transplant Characteristics

Baseline characteristics were compared for contralateral vs first SLT recipients with *χ*^2^ and Fisher exact tests for categorical variables and Wilcoxon rank sum test for continuous variables. Median (interquartile range) was reported for continuous variables, and number (percentage) was reported for categorical variables.

### Posttransplantation Outcomes

This study compared the post–lung transplantation outcomes by *χ*^2^ testing. Length of stay was compared by rank sum testing.

We performed time-to-event analysis with administrative censorship on June 30, 2023, and visualized the incidence of mortality with Kaplan-Meier curves. We used multivariable Cox regression to compare risk of post–lung transplantation mortality for recipients of contralateral vs first lung transplants, adjusting for donor and recipient characteristics with *P* < .2 on univariate analysis, reported as the adjusted hazard ratio and 95% CI.

### Organ Utilization

To evaluate organ utilization, we analyzed offer data for lung transplants from 2015 to 2022 and collected data on offer timing, transplant type, and offer order.

Statistical analyses were performed using R statistical software version 3.6.2 (R Foundation for Statistical Computing) within RStudio statistical software version 1.2.5033 (RStudio).

## Results

### Donor, Recipient, and Transplant Characteristics

Of 4692 SLT recipients, 1246 (26.6%) received a contralateral lung; 3446 (73.4%) received a first lung. Compared with first lung recipients, contralateral lung recipients were more likely to be female (486 [39.0%] vs 1146 [33.3%]; *P* = .003; Table 1). Contralateral recipients were also more likely to be undergoing transplantation for obstructive lung disease (369 [29.6%] vs 685 [19.9%]; *P* < .001) and specifically chronic obstructive pulmonary disease or emphysema (344 [27.6%] vs 661 [19.2%]; *P* < .001). Recipients of a contralateral lung had lower rates of prior lung transplant (28 [2.2%] vs 145 [4.2%]; *P* = .002).

**Table 1:** Baseline Donor, Recipient, and Transplant Characteristics for Single-Lung Transplant Recipients From 2015 to 2022

Variable	Contralateral Lung (n = 1246)	First Lung (n = 3446)	*P* Value
Donor			
Age, y	34.5 (25-48)	34 (24-47)	.18
Cause of death			.18
Anoxia	394 (31.6)	1108 (32.2)	
Cerebrovascular/stroke	358 (28.7)	874 (25.4)	
Head trauma	469 (37.6)	1379 (40)	
Other	25 (2.0)	85 (2.4)	
Sex, female	440 (35.3)	1191 (34.6)	.66
Cigarette use (>20 pack-years)	65 (5.2)	214 (6.2)	.56
Recipient			
Age, y	66 (62-70)	66 (62-70)	.43
Sex, female	486 (39.0)	1146 (33.3)	.003
Race			.04
Black	82 (6.6)	229 (6.6)	
White	1027 (82.4)	2743 (79.6)	
Other	137 (11)	474 (13.8)	
BMI, kg/m^2^	26.7 (23.5-29.6)	26.5 (23.5-29.3)	.24
Serum creatinine, mg/dL	0.8 (0.7-1)	0.8 (0.7-1)	.06
Total bilirubin, mg/dL	0.4 (0.3-0.6)	0.4 (0.3-0.6)	.77
Diagnosis			<.001
Obstructive lung disease	369 (29.6)	685 (19.9)	
Pulmonary vascular disease	16 (1.3)	59 (1.7)	
CF and immunodeficiency	0 (0)	5 (0.1)	
Restrictive lung disease	861 (69.1)	2697 (78.3)	
COPD/emphysema	344 (27.6)	661 (19.2)	<.001
Prior lung transplant	28 (2.2)	145 (4.2)	.002
Prior thoracic operation	137 (11)	431 (12.5)	.18
ECMO	4 (0.3)	25 (0.7)	.18
FEV_1_, % predicted	45 (27-61)	45 (30-59)	>.9
Time on waitlist, d	58 (21-166)	44 (14-125)	<.001
Ischemia time, h	4.6 (3.8-5.5)	4.2 (3.5-5.2)	<.001
DCD	36 (2.9)	171 (5.0)	.003
LAS	36.9 (33.5-42.2)	40.4 (35.3-49.8)	<.001
Sequence No.	26 (11-65.8)	8 (3-25)	<.001
Laterality			.16
Left lung	680 (54.6)	1798 (52.2)	
Right lung	566 (45.4)	1648 (47.8)	

Continuous variables are reported as n (%). Categorical variables are reported as median (interquartile range).

BMI, body mass index; CF, cystic fibrosis; COPD, chronic obstructive pulmonary disease; DCD, donation after cardiac death; ECMO, extracorporeal membrane oxygenation; FEV_1_, forced expiratory volume in 1 second; LAS, Lung Allocation Score.

Contralateral lung recipients had longer waitlist times (58 [21-166] days vs 44 [14-125] days; *P* < .001), longer ischemia times (4.6 [3.8-5.5] hours vs 4.2 [3.5-5.2] hours; *P* < .001), lower median LAS (36.9 [33.5-42.2] vs 40.4 [35.3-49.8]; *P* < .001), and higher median sequence number (26 [11-65.8] vs 8 [3-25]; *P* < .001). Contralateral recipients were less likely to receive organs donated after cardiac death (36 [2.9%] vs 171 [5.0%]; *P* = .003).

### Posttransplantation Outcomes

Length of stay was shorter for contralateral lung recipients (14 [10-21] days vs 15 [11-25] days; *P* < .001; Table 2).

**Table 2:** Transplantation Outcomes for Single-Lung Transplant Recipients From 2015 to 2022

Variable	Contralateral Lung (n = 1246)	First Lung (n = 3446)	*P* Value
Airway dehiscence	14 (1.1)	41 (1.2)	.78
Dialysis	43 (3.5)	164 (4.8)	.16
Acute rejection	85 (6.8)	245 (7.1)	.78
Length of stay, d	14 (10-21)	15 (11-25)	<.001
Pacemaker	3 (0.2)	11 (0.3)	.48
Reintubation	154 (12.4)	507 (14.7)	.13
Stroke	17 (1.4)	69 (2.0)	.50

Continuous variables are reported as n (%). Categorical variables are reported as median (interquartile range).

Survival was higher in contralateral vs first SLT recipients (median survival, 5.0 [95% CI, 4.7-5.3] years vs 4.5 [95% CI, 4.2-4.7] years; log-rank *P* < .001; Figure). This difference persisted as a 14% decreased hazard of mortality for contralateral recipients after adjustment (adjusted hazard ratio, 0.86 [0.77-0.95]; *P* = .004; Table 3).

**Figure:**
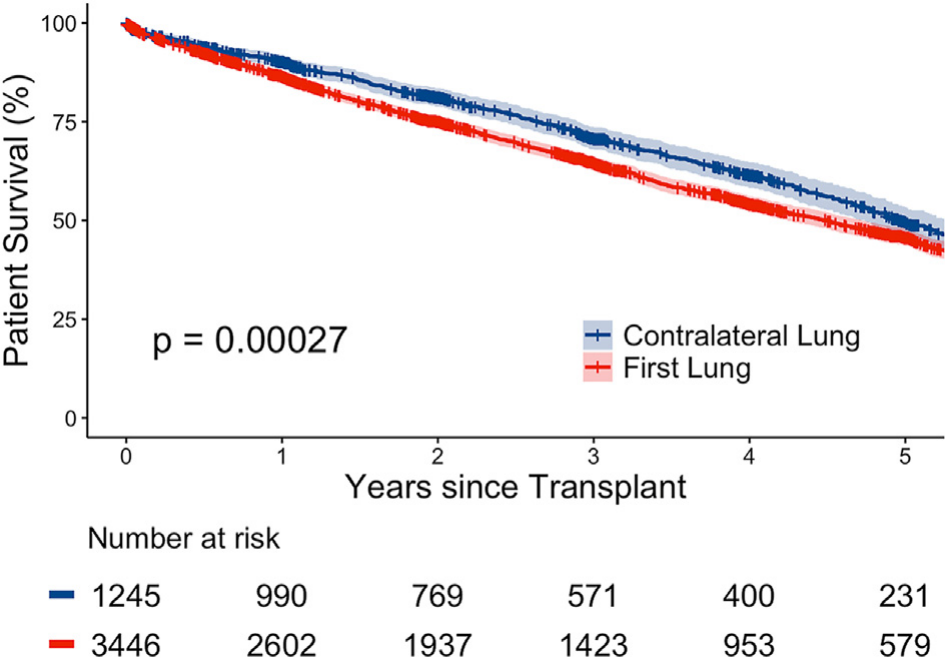
Kaplan-Meier survival curve for single-lung transplant recipients from 2015 to 2022. Survival time for single-lung transplant and 95% CI.

**Table 3:** Multivariate Cox Regression for Posttransplantation Survival in Single-Lung Transplant Recipients From 2015 to 2022

Variable	Adjusted Hazard Ratio	95% CI	*P* Value
Diagnosis			
Obstructive lung disease	Reference		
Pulmonary vascular disease	1.00	0.65-1.52	.99
CF and immunodeficiency	0.77	0.11-5.62	.8
Restrictive lung disease	0.76	0.59-0.98	.04
Laterality			
Left lung	Reference		
Right lung	0.9	0.83-0.98	.02
DCD	0.96	0.76-1.21	.73
FEV_1_	1	1-1	.58
Time on waitlist, mo	1.00	0.99-1.00	.34
Sequence No. (per 10)	0.99	0.98-1.00	.19
Donor age	1.01	1.00-1.01	<.001
LAS	1.01	1.00-1.01	<.001
Recipient age	1.03	1.02-1.03	<.001
Ischemia time, h	1.03	1.01-1.06	.01
Total bilirubin	1.06	1.03-1.08	<.001
Male sex	1.1	0.99-1.21	.06
Diagnosis: COPD	1.11	0.87-1.42	.39
ECMO	1.11	0.58-2.1	.76
Transplant type			.004
First lung	Reference		
Contralateral lung	0.86	0.77-0.95	
Creatinine	1.17	1.05-1.3	.01
Prior lung transplant	1.83	1.46-2.29	<.001

Variables with a *P* < .2 on univariate analysis included in the final multivariate model, which yielded the variables shown.

CF, cystic fibrosis; COPD, chronic obstructive pulmonary disease; DCD, donation after cardiac death; ECMO, extracorporeal membrane oxygenation; FEV_1_, forced expiratory volume in 1 second; LAS, Lung Allocation Score.

### Organ Utilization

Of 20,201 lung donors from 2015 to 2022, 3504 (17.3%) were lung donors to SLT recipients. Of these SLT donors, 1309 (37.4%) donors had both lungs accepted for use. Of these 1309 donors in whom both lungs were accepted for SLT in different recipients, there were 1237 (94.4%) with both SLTs performed and 9 (0.7%) with only the contralateral lung transplant performed.

## Comment

In this national analysis of SLTs, we found that recipients of contralateral lungs were able to undergo transplantation at a lower LAS without compromising posttransplantation survival. Furthermore, the contralateral lung was accepted in only 37.4% of donors.

In this study, we observed similar post–lung transplantation survival for contralateral vs first SLT recipients, despite a significantly higher sequence number. Although some contralateral lungs may not be usable (eg, because of trauma or infection), the low use of the contralateral lungs seen here suggests that there are other reasons for contralateral lung decline. One potential reason could be high sequence number, making these organs perceived as less desirable. However, multiple studies have shown that high sequence number alone is not a risk factor for post–lung transplantation mortality.^7^ For donor organs that are considered marginal for other reasons, the rising use of technologies such as ex vivo lung perfusion might also allow further evaluation and enable greater use of contralateral lungs for single-lung transplantation.^4^^,^^8^

The increased survival observed for contralateral SLTs may reflect transplantation at an earlier and healthier time in disease course. Recipients in the contralateral group were more likely to have chronic obstructive pulmonary disease/emphysema as their diagnosis for lung transplantation, which may progress more indolently with improved post–lung transplantation survival compared with other diagnoses.^9^ Contralateral recipients also had a lower LAS, which has been associated with improved post–lung transplantation survival.^10^ Thus, although our analysis adjusted for baseline recipient characteristics including diagnosis and LAS, there may still be unmeasured confounding creating a healthier contralateral single-lung transplantation group. However, these results suggest that contralateral lung transplantation is not detrimental to post–lung transplantation outcomes and may even allow candidates to access transplantation while they are healthier to achieve equivalent or superior post–lung transplantation outcomes.

### Limitations

This study has several limitations. First, although the use of a national database provides the broadest sample to include, it limits the granularity of the data. Variables like LAS, pulmonary function test results, and diagnosis provide some measure of disease severity, but their imprecision can result in unmeasured confounding. Future studies evaluating the effects of more granular disease severity as well as waitlist outcomes in this population will be of interest. Next, despite the recent change from LAS to Composite Allocation Score (CAS), much of the CAS is made of variables from the LAS, meaning that these findings can still be valuable to providers in this new setting. Reevaluation in the CAS era will be helpful in translating these findings. Finally, future investigations focusing on reasons for discard and the survival impact of turning down a contralateral lung would be of interest to further guide the use of contralateral lungs.

### Conclusion

In this national study of SLTs, the use of the contralateral lung enabled transplantation at a lower LAS compared with first lung recipients, without compromising posttransplantation survival or complications and providing a promising avenue to explore in the effort to expand the donor pool.
